# Analyzing Plant Low-Molecular-Weight Polar Metabolites: A GC-MS Approach

**DOI:** 10.3390/plants15030445

**Published:** 2026-01-31

**Authors:** Tatiana Bilova, Nadezhda Frolova, Anastasia Orlova, Svetlana Silinskaia, Akif Mailov, Veronika Popova, Andrej Frolov

**Affiliations:** 1 Department of Plant Physiology and Biochemistry, Saint Petersburg State University, 199034 Saint Petersburg, Russia; t.bilova@spbu.ru (T.B.); veronika.chantseva@gmail.com (V.P.); 2 Laboratory of Analytical Biochemistry and Biotechnology, K.A. Timiryazev Institute of Plant Physiology, Russian Academy of Sciences, 127276 Moscow, Russia; lanas_95@mail.ru (A.O.); svetlanasilsv@mail.ru (S.S.); 3 Laboratory of Bioanalytical Chemistry, Higher School of Living Systems, Immanuel Kant Baltic Federal University, 236041 Kaliningrad, Russia; afmailov@gmail.com

**Keywords:** primary metabolites, GC-MS-based profiling, plant metabolomics

## Abstract

Decades ago, the introduction of GC-MS marked a significant advancement in primary plant metabolite studies. Here, in our review, we will delve into critical aspects of the workflow, spanning the selection of an analytical platform, sample preparation, analytical acquisition, and data processing and interpretation. The exceptional separation capabilities of GC, characterized by remarkable chromatographic resolution, render it ideal for analysis of the complex plant metabolome, including the separation of isomeric compounds. The diversity of analytical platforms allows the investigation of plant metabolomes using targeted and non-targeted approaches. GC-MS, equipped with efficient extraction methods and reliable derivatization protocols for semi- and non-volatile compounds, enables qualitative and quantitative analysis of these molecules. The stability of derivatives forms the foundation for the robustness and reproducibility of GC-MS methods, and their mass spectra provide characteristic fragments for confident identification and sensitive quantification of individual metabolites. There has been key progress in the advancement of GC-MS approaches to studying plant metabolism. However, the presence of artifacts during GC-MS analysis, particularly during derivatization, is a challenge that requires careful validations, which frequently necessitate additional investigations. The feasible solutions that were achieved to overcome the limitations in GC-MS-based studies are a particular focus of the present discussion.

## 1. Introduction

More than two decades ago, GC-MS was introduced as a method for comprehensive analysis of plant primary metabolites. The initial studies of Roessner et al. (2000) [1] and Fiehn et al. (2000a) [2] utilized gas chromatography–quadrupole mass spectrometry with electron ionization (GC-EI-Q-MS) for untargeted metabolic profiling of polar and semi-polar metabolites in aqueous methanolic and chloroform–methanolic plant extracts, respectively. Roessner and colleagues identified about 150 analytes in polar extracts of potato tubers and showed that the relative contents some of the metabolites (glycerol, sugar alcohols, and amino acids) varied with the plant growth conditions [1]. Similarly, Fiehn et al. analyzed semi-polar and polar metabolites from leaf extracts of different *Arabidopsis thaliana* genotypes. By using principal component analysis (PCA), they successfully distinguished the genotypes based on the relative abundances of 214 polar and 112 lipophilic annotated analytes [2]. These pioneering studies highlighted the potential of GC-MS-based metabolomics in bridging the gap between phenotypes and genotypes and unraveling molecular networks.

Indeed, the plant metabolome can be interpreted as the realization of genetic information, a kind of “bridge” between genotype and phenotype [3]. This understanding stems from the fact that metabolites always play a specific role in the cell and organism, representing the end result of gene function [4]. GC-MS allows the detection of quantitative differences in the metabolite content of plants, which, in combination with data analysis tools, can serve as a method for comprehensive characterization of plant genotypes in response to biotic and abiotic stresses [5,6,7,8], symbiotic interactions [9,10], and oxidative stress [11], as well as during growth, development and ageing [12]. It should be noted that GC-MS has many other cross-border applications, including the search for new biomarkers and bioactive metabolites [13,14], chemotyping [15], metabolomics-based selection and creating “next generations” of agricultural crops [16,17], and metabolic pathway engineering [18].

The wide range of applications of GC-MS is a clear sign of the technique’s high analytical potential, robustness and reproducibility. This method started its development with the first coupling homemade gas chromatograph with a time-of-flight mass spectrometer (TOF-MS) produced by the chemists R. Gohlke and F. McLafferty in the 1950s. The resulting GC-MS demonstrated a revolutionary enhancement in its analytical power [19]. Thus, by combining the exceptional compound-separating strength of GC and the definitive identification capacity of MS, it became possible to analyze each component emerging from the gas chromatograph separately. The first GC-MS instruments revealed many technical challenges, which determined the path for 25 years in the development of this method [19]. Thus, by 1980, GC-MS had achieved significant advances due to innovations in quadrupole (Q)-MS and faster TOF-MS, the evolution of efficient and reproducible fused-silica capillary columns, affordable computers for data processing, and improved electronics. In the following years further GC-MS-based technical innovations such as comprehensive two- and multi-dimensional gas chromatography (2DGC/MDGC)-TOF-MS, GC–time-of-flight tandem mass spectrometry (TOF-MS/MS), GC–ion mobility mass spectrometry (IMS), and GC coupled with a range of high-resolution mass spectrometers (HRMSs) have been developed [20,21,22,23]. Those innovative technologies together with improvements in raw data processing software, mass spectrum libraries, MS search and bioinformatics tools, and, in addition, the consistency of GC with a variety of sample preparation methods and derivatization strategies, have formed a whole GC-MS-based methodology appropriate for analyzing diverse low-molecular-weight compounds with markedly different physicochemical properties, such as non-polar and semi-polar volatiles and semi- and polar non-volatile metabolites.

Nevertheless, despite the rapid development of GC-MS-based innovative technologies, the canonical single-dimension GC-MS remains a routine method in every laboratory and a preferred method in applied and fundamental plant sciences due to its high efficiency in profiling of plant compounds, robustness, and the equipment being relatively inexpensive but reliable [24,25,26,27,28,29]. Thus, GC-MS has been widely used in research to identify metabolites responsible for the biological properties of medicinal plants, e.g., leaves of tropical plants such as *Jacaranda mimosifolia* [30], *Aporosa cardiosperma* [31], and *Senecio scandens* [32]. In this type of study, crude plant extracts usually are not subjected to derivatization. Their GC-MS analysis focuses on the identification of non- or semi-polar volatile secondary compounds (sesqui-, di- and triterpenoids; saturated fatty acid alcohols; and alkaloids) which may exhibit biological activities and appear to be plant-specific [33]. Usually such profiles cover just several dozen metabolites [30,31,32,33].

However, by implementing a derivatization strategy in the GC-MS workflow, the coverage of metabolites can be significantly expanded up to several hundred metabolites [5,34] as it transforms most non-volatile semi-polar and polar molecules dominating in plant extracts into volatile targets for GC-MS. Most metabolites of the groups are participants in major energy and biosynthetic metabolite pathways. Therefore, even though derivatization introduces complexity (potential incompliteness of reactions and formation of several derivatives) leading to the appearance of artifacts [29,35,36,37], this strategy has made GC-MS an apropriate method for analyzing the plant primary metabolome, significantly increasing the method’s versatility. Moreover, this progress in unravelling the plant primary metabolome using GC-MS has been proven to be a significant contribution to multi-omics research. This can be demonstrated by multiple studies in which GC-MS-based metabolite profiles helped in linking genomic and proteomic data to phenotyic traits of interest, enabling metabolomics-assisted beeding for crop improvement [38], and revealing mechanisms of development [39], growth protectors [40], and responses to a changing environment [41]. Thus, by means of GC-MS Acharjee and co-workers [38] identified metabolites associated with carotenoid biosynthesis contributing to potato tuber flesh color. In another work, Wang and co-authors [40] revealed that the growth-promoting effect of zaxinone on rice seedlings was accompanied by a marked increase in the contents of many sugars and stimulating central energy pathways. And in the third example, Yun and colleagues [39] found 36 GC-MS-identified primary metabolites and 81 volatiles that contributed to the ripening of the peel in harvested bananas.

Thus, the obvious advances of GC-MS for analysing polar metabolites can be listed as follows: (i) The outstanding efficiency of GC separation with a chromatographic resolution as high as 100,000 per run [21] is ideal for the extremely complex plant metabolome, often allowing the baseline separation of even isomeric analytes [21]. (ii) Efficient extraction methods, as well as reliable well-established derivatization protocols compatible with analysis of polar compounds, allow the use of GC-MS for the qualitative and quantitative analysis of polar metabolites in plants [42,43]. (iii) The remarkable stability of the derivatives underlies the high robustness and reproducibility of the GC-MS-based methods [44]. Moreover, the electron ionization (EI) spectra of derivatives often yield characteristic highly intense fragments which give access to both (iv) confident identification and (v) sensitive quantification of individual metabolites with a good linear dynamic range (LDR) [45].

To simplify consideration of the complex methodology of GC-MS-based metabolomics, it can be addressed as a generalized workflow comprising three principal steps: (i) sample preparation, (ii) GC-MS analysis and (iii) processing of the acquired data. Since the overall performance and the final output of the metabolomics analysis critically depend on each of these three steps, we address them in more detail below, highlighting their challenges and achievable solutions. However, the importance of the sample preparation, including derivatization methods, in GC-MS-based metabolomics is given special attention because inaccuracy made at this step may distort the final results of the analysis. Numerous sample preparation protocols for GC-MS provide all the necessary information concerning harvesting, freezing, storage, and homogenizing, as well as metabolite extraction, concentration, derivatization, and resuspension [46]. In general, the details of the sample preparation protocol depend on the purpose and object of the study and the choice of the analytical platform [47].

## 2. Choosing an Analytical Platform

Choosing the right analytical platform is a challenge that researchers face at the experiment design stage. The choice depends on the experiment objectives: whether the target metabolites or their groups will be searched for, or whether a non-targeted approach will be used to analyze all metabolites in the sample. Depending on the choice of a targeted or non-targeted approach, both chromatographic separation conditions and mass spectrometry platforms are selected.

In plant metabolomics analysis, when coupling GC-based separations with mass spectrometry, careful consideration of the spectral acquisition rate is essential, as well as understanding the unique capabilities and limitations of each platform, which can impact their suitability for targeted and untargeted metabolomics strategies (Table 1). To ensure reliable peak reconstruction and reproducible peak areas, a minimum acquisition of 7 to 10 data points across the chromatographic peak is recommended [48,49]. For conventional GC separations, a minimum acquisition rate of 2 spectra s^−1^ is sufficient, while GC×GC separations require a minimum acquisition rate of 30 spectra s^−1^ [21]. The choice of mass analyzer significantly impacts the quality of the mass spectrum and the sensitivity of the GC-MS method. Quadrupole mass analyzers are popular in metabolomics due to their versatility and sensitivity. They register the mass spectrum of a compound by scanning the *m*/*z* range (typically from 50 to 700 *m*/*z*) and collecting ions of each *m*/*z* value at one time which pass through the quadrupole sequentially. However, the quadrupoles, as transmission scanning instruments, can experience spectral skewing (i.e., distortion of the mass spectrum due to analyte concentration changes during the scan period, which occurs mainly on the back side of a chromatographic peak) if the acquisition rate is incompatible with the peak width [50]. Faster acquisition rates are necessary to avoid skewing, but they may result in some loss of sensitivity [51]. Non-scanning instruments like time-of-flight (TOF-MS) and Fourier transform (FT-MS) mass spectrometers which acquire the entire mass spectrum simultaneously in a single scan do not exhibit spectral skewing [52,53]. TOF-MS instruments, which perform ion separation and mass assignment based on the velocity of ions as they travel through a flight tube, offer high sensitivity and resolution and operate at acquisition rates between 50 and 200 spectra s^−1^. The Orbitrap mass analyzer separates ions by their axial oscillations in an electric field and detects the ion oscillations, generating an image current which is subsequently Fourier-transformed into a mass spectrum. The Orbitrap provides ultrahigh resolution and accuracy but has an acquisition rate-dependent resolving power [23,54,55]. Untargeted metabolomics (metabolite profiling) requires all features (compound peaks) of mass spectrum acquisition, while targeted metabolomics benefits from improved resolution and additional mass selectivity [45]. Metabolic profiling may also explore precursor and product ion scans for group-type analyses using sequential mass spectrometers, such as triple quadrupole (QqQ-MS) or hybrid Q-TOF-MS. Other scanning modes like selected ion monitoring (SIM) and selected reaction monitoring (SRM) are employed for targeted analysis, offering exceptional selectivity [56]. Extracted ion chromatograms (XICs) can also enhance selectivity, especially with high-mass-accuracy data from TOFMS and Orbitrap instruments (Table 1).

## 3. Sample Preparation

### 3.1. Harvesting and Fixation of Plant Material

**Figure 1:**
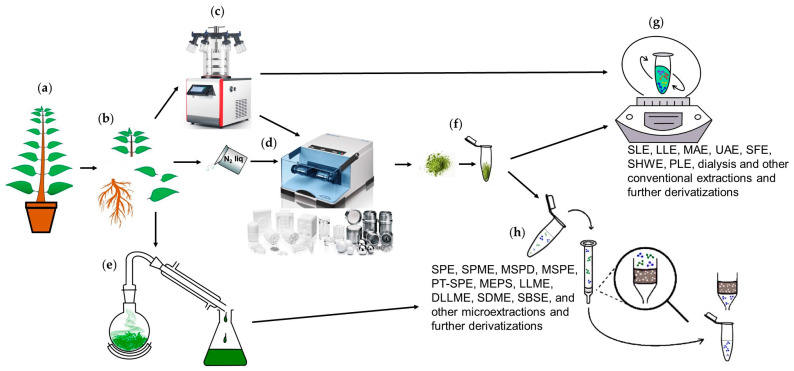
Workflow of sample harvesting, fixation and extraction for GC-MS analysis: (**a**) experimental plant object; (**b**) harvesting; (**c**) freeze drying; (**d**) freezing by liquid nitrogen and grinding by ball mill; (**e**) steam distillation; (**f**) weighing samples; (**g**,**h**) extraction techniques: (**g**) SLE—solid–liquid extraction, LLE—liquid–liquid extraction, MAE—microwave-assisted extraction, UAE—ultrasound-assisted extraction, SFE—supercritical fluid extraction, SHWE—super-heated water extraction, PLE—pressurized liquid extraction; (**h**) SPE—solid-phase extraction, SPME—solid-phase microextraction, MSPD—matrix solid-phase dispersion, MSPE—magnetic SPE, PT-SPE—pipette-tip SPE, MEPS—microextraction by packed sorbent, LLME—liquid–liquid microextraction, DLLME—dispersive liquid–liquid microextraction, SDME—single-drop microextraction, SBSE—stir-bar sorptive extraction.

Harvesting and subsequent homogenization are the first steps of sample preparation (Figure 1) and are important for successful GC-MS analysis [57]. Harvesting of plant material needs to be accomplished rapidly, ideally within 15–30 s and is followed by immediate fixation, e.g., by shock-freezing in liquid nitrogen [29,58,59]. A harvesting delay of up to 30 s is considered a sufficient time to prevent the development of wounding signaling [60,61,62]. Shock-freezing in liquid nitrogen captures the actual metabolome state for each sample at any time point chosen according to the experiment design. Thus, shock-freezing provides a footprint of plant metabolism in the form of a “metabolic snapshot”, preventing any further modification of the sample, such as decomposition of metabolites or changes in their concentration or chemical or physical properties. Since metabolites are highly dynamic (in time and space), such a snapshot helps to clearly assess the current physiological state of the plant at a given time and helps to minimize errors associated with sample preparation [59].

Homogenization techniques serve the essential purpose of obtaining representative samples for analysis by disrupting plant cells. Several homogenization methods are commonly employed in plant metabolomics. One widely used method is mechanical disruption, which involves grinding or crushing plant material using a mortar and pestle, blender, or bead mill [63]. The mechanical methods are effective in breaking down plant tissues and releasing metabolites, but they may generate heat, potentially leading to metabolite degradation or enzymatic reactions [64]. To prevent this, liquid nitrogen or dry ice is used during grinding, as well as cooled mills. Liquid nitrogen freezing effectively preserves the metabolite composition by rapidly halting enzymatic activity and minimizing degradation [59]. However, it requires specialized equipment and poses safety considerations due to the handling of cryogenic substances. Additionally, care must be taken to prevent cross-contamination between samples and ensure consistent and reproducible homogenization [64,65]. For this purpose, separate containers (or tubes) are used for each sample with additionally placed special grinding balls, the main property of which should be inertness [66].

Besides shock-freezing and further grinding, other material fixation methods like microdissection [67] and direct drying [68], as well as fixation techniques combined with extraction methods like microextraction [69], ultrasound-assisted extraction [70], and microwave-assisted extraction [71], showed their excellence in many metabolomics studies, e.g., metabolic profiling [29,65] and identification of biomarkers [72,73] and compounds with potential antimicrobial and antioxidant activities [31,74].

Various microdissection techniques (manual microdissection, laser microdissection, and microsampling) enable the precise isolation of specific plant tissues or cells, facilitating the study of metabolites within targeted structures and cell types, thereby providing valuable insights into their metabolic profiles and functions [54,75]. In the first technique, microtools and a microscope are used; in the second, laser technology is used; and in the third, fine capillaries or microsampling devices are used to carefully isolate specific plant tissues, cell clusters or even individual cells [76,77]. However, the limitations of microdissection are that these procedures can be time-consuming and labor-intensive, especially when targeting small or delicate plant structures. Challenges also may arise in precisely isolating specific cells or tissues due to their complex anatomical arrangements or the potential loss of metabolites during the dissection process [78,79].

Plant samples can also be analyzed in their dried form, enabling the determination of specific components based on their dry mass and significantly mitigating issues related to the high water content in crude samples. Importantly, drying plant material does not completely eliminate water, and the term “dry mass” indicates that the material still contains certain percentages (from several to a dozen(s)) of water [63]. Drying of plant material is often carried out by freeze-drying (also known as lyophilization), where water in the form of ice at low pressure is removed from the material by sublimation or by hot air (at 70–80 °C or lower temperatures, such as 40–50 °C, which is particularly important for relatively volatile or subliming analytes) in ventilated ovens or in ovens with a flow of nitrogen. In cases where non-volatile and non-subliming substances need to be isolated, laboratory vacuum ovens are equipped with water absorption, adsorption, or freezing-out systems. However, vacuum systems are not suitable for highly or even medium-volatile substances.

Some types of the combined fixation–extraction and various extraction techniques applied in GC-MS-based metabolite analysis have already been discussed in our recent work [47] and are treated in other well thought-out reviews on this topic [78,80]. An overall workflow of the sample preparation steps in GC-MS analysis addressed here (harvesting, fixation and extraction) is illustrated in Figure 1, which summarizes the variety of applied methods.

### 3.2. Extraction of Primary Metabolites

High efficiency and selectivity are the main prerequisites for a successful extraction method. Thus, on the one hand, an efficient extraction procedure yields complete isolation of the desired metabolite groups; on the other, the co-extraction of proteins, polysaccharides, nucleic acids and lipids needs to be avoided. As plant primary metabolites vary essentially in their physicochemical properties (polarity, pKa, volatility, and termostability) and relative abundances (by up to several orders of magnitude), their concerted and efficient extraction is challenging. Therefore, selection of the apropriate solvent systems (in terms of polarity and compatibility with the target analytes) is critical [81]. To isolate all metabolites containing polar groups, polar solvents like methanol [82,83,84], isopropanol [85], and alcohol-aqueous solutions [16,86,87] are commonly used in various studies [88,89,90,91]. In addition to single-stage extraction with one or a mixture of solvents, multi-stage methods are often used to enhance the coverage and efficiency of metabolite extraction. These multi-stage extraction approaches involve a series of different solvents or solvent mixtures in sequential steps to target specific groups of metabolites and improve their overall yield [61]. In the first stage, less polar solvents (isopropanol, chloroform, and methanol) are typically added to the plant material, facilitating the extraction of lipids, waxes, and other non- or semi-polar metabolites. In the second stage, polar solvents like water are used to extract polar metabolites (sugars, organic acids, and amino acids). This two (or more)-stage procedure allows more comprehensive extraction of metabolites of varying polarities, which may be analyzed as a complex or as a separate extracts [61].

To access higher extraction efficiency, additional approaches are utilized to optimize the solubility and stability of metabolites, e.g., by manipulating the temperature conditions [81,92]. Temperature optimization is crucial to achieve a comprehensive and representative metabolite profile [57,58]. Despite enhanced temperatures (70–85 °C) providing better solubility for many polar compounds, lower temperatures can be advantageous to get better recoveries of more volatile compounds. Additionaly, lower extraction temperatures favor the stability of chemically labile metabolites, minimizing the potential for their degradation and side reactions during the extraction process. In this context, sample homogeneity should also be considered, as less homogeneous samples might require longer extraction times, which makes higher temperatures beneficial for efficient extraction [93].

To ensure the optimal separation efficiency, the analyzed samples need to be free from the metabolites, which are incompatible with the selected chromatographic system. As compounds cannot be efficiently retained in the stationary phase or/and eluted from it, their adequate quantification appears impossible [21]. Moreover, these contaminants can overload or even damage the separation column, which might compromise the whole metabolomics experiment [81]. In particular, the use of methanol for extraction of polar metabolites suggests that triglycerides and phospholipids are additionally extracted from the plant material with relatively high yields. Because of their relatively high molecular weight, these lipids and their trimethylsilylated derivates (as well as high-weight products produced due to the thermal instability of the derivates [35]) remain non-volatile under the high-temperature conditions used to introduce a liquid sample in a GC system and, therefore, will be retained on walls of the glass liner and may bind irreversibly with poly(dimethyldiphenylsyloxane) phases that are commonly used in GC analysis [21]. Moreover, the lipids accumulated on the liner may be subjected to at least partial pyrolysis under the high-temperature injection conditions [94]. This might result in the increasing intensity of background signals related to fatty acids and their degradation products, which is often also associated with their strong carry-over. Indeed, this phenomenon is often observed as contaminant signals of saturated fatty acids like palmitic and stearic acids [94].

To overcome the above-described issue and to ensure the adequate quality of the quantitative data, metabolites such as lipids, waxes and other high-molecular-weight non-polar compounds which cannot be efficiently analyzed in a system designed to separate low-weight volatile compounds need to be removed from the extract prior to the GC-MS analysis, e.g., by liquid–liquid extraction using non-polar solvents such as n-hexane or n-heptane. Indeed, Fiehn and co-workers showed that removal of lipids significantly increases the accuracy of the analysis [94]. Removal of lipids also essentially improves the efficiency of amino acid and polyamine subsequent derivatization (trimethylsilylation) [45,87].

As multiple factors might affect extraction performance, the reproducibility of this sample preparation step is important and requires special attention. Thus, in metabolomics, control of the extraction performance relies on internal standardization [45,87]. An internal standard (IS) is supplemented to the extraction solvent to consider any potential analyte losses accompanying the whole sample preparation procedure, including besides extraction such steps as extract drying, storage and derivatization [45,81]. Typically, non-natural compounds (at least those not found in plants of interest) with a structure and analytical behavior similar to the target analytes are selected as ISs [45]. For analysis of primary metabolites, the following standards are often used: ribitol (adonitol) [1,86,88], lactose [2,95], or stable isotope-labeled compounds such as [1,2,3-^13^C_3_]myristic acid [96].

### 3.3. Derivatization

The volatility and thermal stability of the analyzed substances are the key prerequisites for successful GC-MS analysis [45,81]. Due to the presence of polar or even charged functional groups, primary plant metabolites are typically not volatile and cannot therefore be efficiently introduced in GC by liquid injection [21]. To adjust the physical properties of these analytes to the requirements of the GC-MS methodology, the polar functional groups (amine, hydroxyl, carboxyl, and thiol groups) of the prospective analytes need to be “caped” by non-polar moieties. This chemical modification of polar functional groups can be achieved by a broad array of derivatization methods which have already been discussed by us in our previous review on sample preparation and derivatization methods [47] and in more detail in the comprehensive review of Evershed and Beale et al. [92].

If performed correctly, this derivatization of polar plant metabolites results in a quantitative transfer of the resultant derivatives to the gaseous phase in the liquid injector of the GC. Thereby, the hydrophobicity of the resultant derivatives allows their efficient retention in non-polar stationary phases, such as the commonly used poly(dimethylsyloxane) and poly(dimethyldiphenylsiloxane), and complete elution from them by reasonable temperature gradients [21].

When choosing the derivatization strategy, the chemical nature and structure diversity of the target analytes must be taken into account. Since trimethylsilylation [97] and its modification—*tert*-butyldimethylsilylation [98]—exhibit high reactivity towards practically all polar functional groups of most plant metabolites, these derivatization strategies are recognized as the most universal and suitable in primary metabolomics [5,31,99,100]. Both methods rely on the reaction of sylilation, i.e., the substitution of acidic protons of polar groups with organosilicon radicals—trimethylsilyl ((TMS)_3_Si^•^) and *tert*-butyl(dimethyl)silyl (TBDMS) [35]. The resultant trimethylsilyl (TMS) derivatives are well soluble in non-polar organic solvents (chloroform, hexane, and dichloromethane) and demonstrate low reactivity, high volatility and thermal stability [35]. *N*-methyl-*N*-(trimethylsilyl)trifluoroacetamide (MSTFA) and *N*,*O*-bis(trimethylsilyl)trifluoroacetamide (BSTFA) are the most widely used and efficient among commercially available derivatization reagents due to their capacity to quickly derivatize polar groups of metabolites, leading to improved stability and better sensitivity of the resulting trimethylsilyl derivatives in GC-MS analysis [45]. Fortunately, the trimethylsilylation reaction is typically accomplished at moderate or even ambient conditions (temperature range: 30–90 °C; time range: 0.5–2 h) [29,97]. Other silylation agents, such as *N*,*O*-bis(trimethylsilyl)acetamide (BSA), *N*-trimethylsilylacetamide (TMSA), and *N*-trimethylsilylimidazole (TMSI), can also be used for derivatization in metabolomics studies. However, they are not widely used due to their lower reactivity and less efficient silylation process. Trimethylsilylation, despite its high efficiency and multiple advantages, has several intrinsic limitations. Trimethylsilylation agents and TMS derivatives are highly prone to hydrolytic degradation, and therefore they are extremely sensitive, even to traces of water [21]. In the presence of moisture, the agents are decomposed, producing *N*-methyltrifluoroacetamide and hexamethyldisiloxane. Therefore, prior to derivatization, the extracts need to be thoroughly dried. Our experience indicates that for dried extracts, additional short (20 min) drying under reduced pressure directly prior to derivatization essentially improves the reproducibility of the analysis (unpublished observation). On the other hand, this high water sensitivity can be overcome by application of *N*-*tert*-butyldimethylsilyl-*N*-methyltrifluoroacetamide (MTBSTFA) and *N*-methyl-bis(trifluoroacetamide) (MBTFA), due to their experimentally discovered lower susceptibility to hydrolysis, making them more suitable for samples with higher water contents or greater water sensitivity [101,102]. The increased stability of *tert*-butyldimethylsilyl (TBDMS) derivatives allows for a more accurate and reliable analysis of compounds, especially those prone to degradation in the presence of water. However, due to the increase in the number of carbons of the derivatization reagent, TBDMS derivatives would have different retention times (t_R_s) and retention indices (RIs) relative to TMS derivates, and the analysis time would be increased [103].

One of the crucial steps in preparing samples for GC-MS analysis is the use of *O*-methylhydroxylamine hydrochloride (MeOX) for derivatization. This derivatization helps to reduce the number of spatial isomers of sugars from five to two and thereby increases the accuracy of sugar identification [47,104]. Subsequent (i.e., combined in a two-step process) derivatization with MeOX and trimethylsilylation is a common practice in plant metabolomics, employed to achieve a comprehensive GC-MS analysis of metabolites. Firstly, MeOX is dissolved in pyridine (which serves as the catalyst of the subsequent sylilation reaction) used for the derivatization of selective targets such as sugars and transforms them, increasing their volatility and stability. Then, trimethylsilylation further improves the stability and volatility of the sugars and a wide range of other polar metabolites, making them compatible with GC-MS analysis. This protocol for the combined derivatization of polar metabolites proved to be efficient in a wide range of plant metabolomics studies, including the metabolic profiling of a wide range of plant species (*A. thaliana*, *R. sativus*, *A. caudatus*, *B. napus*, *P. sativum*, etc.) [5,99,105,106], and in studies devoted to alternations of the primary metabolome caused by plant aging, an impact of stress [5,107], and in vitro formation of glycation reaction products [108].

However, the combined MeOX and TMS derivatizations are not without limitations. MeOX derivatization is typically conducted under an elevated temperature (30 °C) and requires an extended period of time (1–2 h), which may introduce artifacts in crude samples, such as an increase in relative contents of pyroglutamic acid (also known as 5-oxoproline) and phosphoric acid, most probably because of spontaneous cyclization of glutamic acid and decay of organic phosphates (sugar phosphates), respectively, leading to potential misinterpretations of the results [36]. TMS derivatization, in turn, is accompanied by the appearance of various by-products which potentially might coelute with analyzed metabolites. Detailed structural annotation and mass spectrum description of the silylation by-products are given by Little [109]. Secondly, silylation reaction exhibits higher reactivity towards carboxyl and hydroxyl groups than amino groups. This leads to partial incomplete derivatization of amino groups in molecules of amino acids [35]. As a result, trimethylsilylation of amino acids usually results in the formation of several (2–4) amino acid derivatives with different numbers of TMS groups [29,37,97,110]. Moreover, TMS derivatization of certain amino acids can lead to a change in their structure and the formation of a different amino acid. This can be exemplified by arginine derivatization, which could lead to the formation of Ornithine lactam 2TMS, Ornithine 3TMS and Citrulline 3TMS [29,37]. Thirdly, the elevated temperatures at which silylation usually takes place can cause the destruction of thermally unstable compounds. Therefore, it should be kept in mind that some compounds detected on chromatograms may be method-related artifacts, i.e., they may be the breakdown products of thermolabile compounds. Finally, attention must be given to the derivatization of crowd samples with high amounts of sugars and/or organic acids, due to their differences in pKa, which could result in differences in derivate yields [97]. Thus, careful validation and consideration of potential artifacts are essential when applying MeOX and TMS/TBDMS derivatization in plant metabolomics studies, and further investigations of this problem are needed.

Fatty acids, a vital metabolite class of plants, play essential roles in various cellular processes, including energy storage and membrane structure, and serve as precursors for signaling molecules and secondary metabolites, making their analysis of significant interest in plant metabolomics studies [111,112]. However, due to their low volatility and poor ionization efficiency, the direct analysis of fatty acids by GC-MS is challenging. To address this issue, as well as silylation, another commonly used derivatization reaction involves converting fatty acids in the reaction of transesterification with a methanol into more volatile and easily detectable derivatives known as fatty acid methyl esters (FAMEs). The reaction requires a strong acid catalyst, such as boron trifluoride (BF_3_) or sulfuric acid, and elevated temperatures to ensure the complete conversion of fatty acids into FAMEs [35,113,114]. The fatty acid derivatization into FAMEs offers the following advantages: (i) increased volatility, which allows for shorter retention times and better chromatographic separation, leading to improved sensitivity and resolution [35]; (ii) enhanced ionization efficiency, which results in higher-quality mass spectra and reliable identification of fatty acid species [45]; (iii) a broader range of analyzable fatty acids, including those with varying chain lengths and degrees of unsaturation. This versatility is essential for profiling the diverse fatty acid composition and elucidating the roles of different fatty acids in the physiological processes of plants [114,115].

Fatty acid derivatization into FAMEs has some limitations. The derivatization may result in incomplete conversion of fatty acids into their corresponding derivatives [35], leading to the underestimation or misidentification of certain fatty acids, and may introduce artifacts or chemical modifications that could potentially affect the interpretation of the results [94]. The efficiency of the transesterification can be influenced by reaction time, temperature, and the catalyst concentration. To minimize the potential for incomplete derivatization and, therefore, to ensure reproducibility and consistency across samples, it is crucial to optimize the reaction conditions [113].

Overall, the derivatization of polar compounds greatly expands the GC-MS analytical capabilities. Indeed, due to derivatization, polar and non-volatile compounds obtain their volatility, chemical and thermal stability in the form of the resultant derivates. Thus, derivatization enables GC-MS to perform robust profiling of primary metabolites in plant samples, enhancing our understanding of plant metabolism in general and the roles of specific metabolites in altering plant metabolism during various physiological processes.

## 4. Analytical Acquisition

GC-MS analytical acquisition can be performed in scanning, selected ion monitoring (SIM), scan/SIM, selected ion recording, selected ion storage, tandem mass spectrometry (MS/MS), multiple reaction monitoring (MRM), selected reaction monitoring (SRM), and multistage mass spectroscopy (MS^n^) modes [116]. In scan mode, which is commonly used in untargeted and targeted analyses of MEOX-TMS derivatives of polar plant metabolites, a range of ions such as *m*/*z* 50–700 is often selected, and continuous change in the voltages on the quadrupole mass spectrometer allows scanning over this predetermined range. In the results of a GC-MS analysis, a total ion chromatogram (TIC) is built. This presents a plot of the sum of total ion intensity in the given *m*/*z* range as a function of time. The TIC is built as two dimensions of data. Each point on the TIC contains a mass spectrum, which is a distribution of the relative intensity of ions acquired during a scan. A mass spectrum acquired at the top of the TIC peak or an average of the mass spectrum collected across the TIC peak is often used to identify the analyte by comparison with its spectrum and comparison of the RI with the parameters from the MS library. In SIM mode, which is often used in targeted analysis, only a few ions of interest are selected to greatly increase the sensitivity of the mass analyzer to them. This is achieved by setting the quadrupole voltage to scan only one or a group of the ions. SIM mode improves the signal-to-noise ratio (S/N) because the analyzer is able to spend more time collecting specific ions while reducing noise. Selecting the specific ion to analyze in SIM mode requires prior knowledge of the mass spectrum of the compound, which in turn can be obtained by analyzing the standard of interest in scan mode. Any issues which can impact instrument sensitivity, such as extract degradation, column contamination, and t_R_ shifts, would affect the data acquisition results. Therefore, to ensure high-quality control in targeted and untargeted GC-MS metabolomics, several of the following recommendations should be followed. The condition of the chosen analytical platform should be regularly (ideally before every analysis of experimental samples) evaluated according to standard protocols described in the instrument documentation and/or the scientific literature [117]. This is necessary to ensure that there are no changes in instrument sensitivity that are usually observed during a metabolomic profiling analysis due to contamination of an injector, an analytical column and an ion source by non-volatiles, as well as column degradation which can also cause shifts in analyte t_R_s. This evaluation involves analyzing quality control samples (QCs) or mixes of standards. Particular attention should be paid to the content of amino acid TMS derivatives because these compounds are very susceptible to degradation during GC-MS analysis. In amino acid TMS derivates, the ratio of nitrogen–silicon bond formation and decomposition strongly depends on the clearness of the injector (liner, syringe needle, and gas line) and the column [21,45]. To assess the potential losses of the derivatives, and, therefore, the clearness of the GC system as a whole, it is recommended to monitor on a daily basis the relative abundance of the amino acid TMS compounds in relation to carbohydrate TMS derivatives [45]. Additionally, to maintain chromatographic resolution, method development should also consider the maximum number of injections per GC column [21]. For large-scale plant metabolomics studies, it is important to replace columns and consumables before encountering quality issues. It is also important to optimize the analytical load of a GC capillary column with an analyzing sample to avoid the column overloading. For that, sequential GC-MS analysis of increasing sample doses is performed. Then, the optimal sample dose (i.e., the sample quantity for GC-MS analysis which does not cause column overload) is calculated from a linear equation observed for linearity between applied sample doses and peak areas obtained from a corresponding chromatogram.

**Figure 2:**
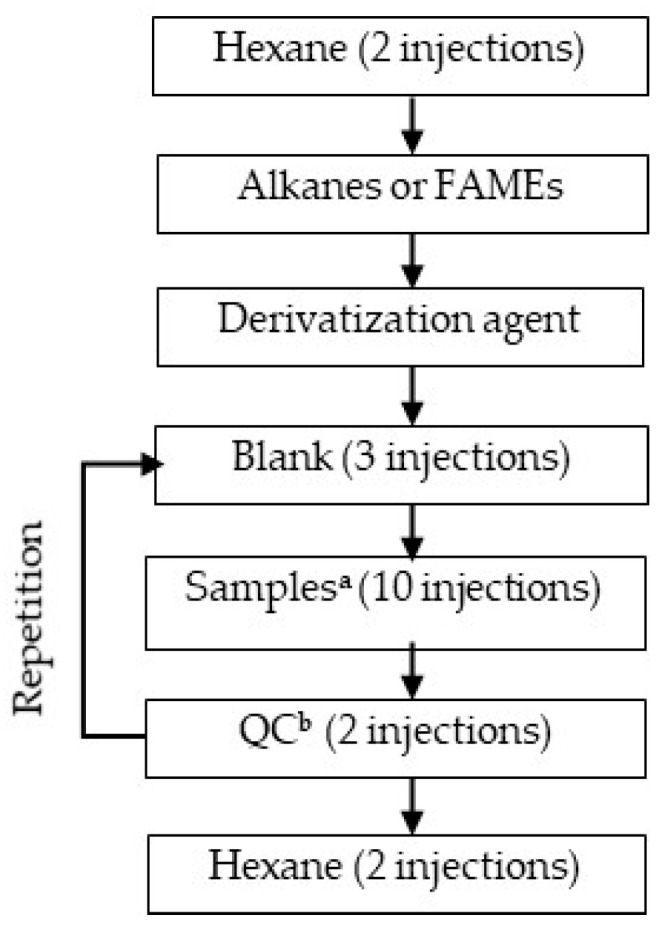
Typical sample sequence (batch) for GC-MS analysis. Mixes of alkanes or FAMEs are used for RI calibration. ^a^ Experimental samples (randomly arranged), mix of standards which can be reference compounds, external calibration or standard addition calibration (n ≥ 3); ^b^ at least 6 separate QC samples in duplicates during the analysis [45].

Finally, several recommendations for designing sample sequences (batches) are as follows: (i) arrange hexanes, blanks, a mix of alkanes, metabolite standards and QCs in the same batch as the experimental samples; (ii) randomize the experimental samples across the batch; (iii) place up to six QCs in different positions across the batch (Figure 2).

Including blanks in a GC-MS sample sequence is needed to monitor possible contamination which might occur during GC-MS analysis. Blanks contain solvents used in sample preparation but without the analytes of interest. They are processed along with experimental samples (Figure 2). Contamination in blank chromatograms is indicated as so-called “ghost peaks”, i.e., peaks that are not expected to be in a chromatogram, and background (noise) signals [57]. Examining the mass spectra of the ghost peaks found in blanks helps to identify potential contamination sources such as reagents and solvents, vial septa, injection septa, liners, and columns [118]. Thus, monitoring contaminations with blanks helps to detect ghost peaks and exclude them from obtained sample-related GC-MS data, leading to more robust interpretations of metabolomic profiles. Additionally, the introduction of a derivatizing agent in a GC system before blanks allows column conditioning, during which residual contaminants are flushed out, making the column fit for reliable use [102]. Linear alkanes are included in GC-MS analysis to be used in t_R_ calibration, allowing for consistent alignment and matching of analyte t_R_s across different GC-MS runs [119].

QC samples, usually obtained by mixing equal aliquots of each biological sample are essential in assessing within- and between-series repeatability and removing features with excessive signal drift prior to statistical analysis. They are also used for equilibrating the analytical platform, checking metabolite profiles, calculating technical precision, signal correction, and standardization [57,87].

Standards of expected metabolites are included in sample batch and serve as both reference compounds for accurate identification of detected analytes and for their absolute quantification [57,87]. Ideally, the standards should cover a wide range of chemical properties and concentrations to match the diversity of the analytes present in the sample. The selection of standards usually relies on the literature data and the personal experience of a researcher in working with the biological object under study. Based on this information, a list of expected metabolites of the object under study is compiled.

### Analyte Absolute and Relative Quantification

**Figure 3:**
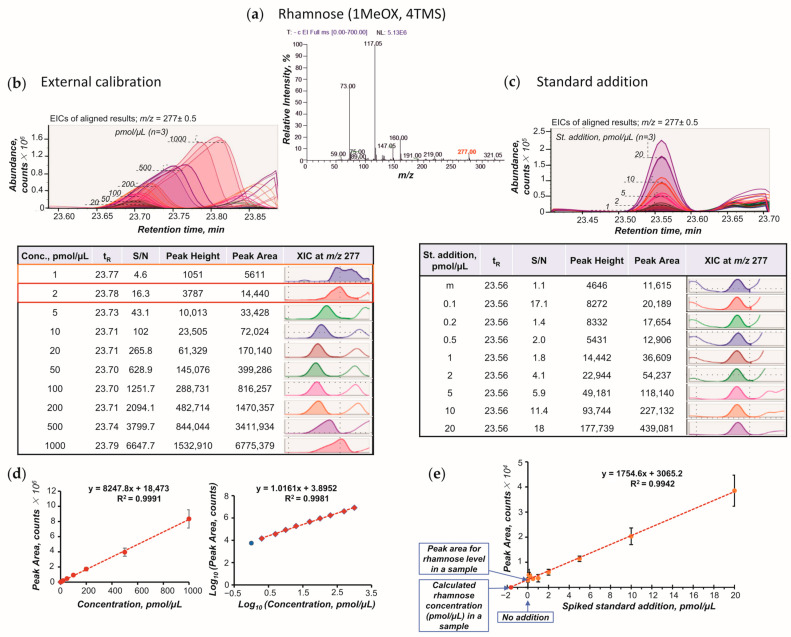
Example of external calibration and standard addition results for rhamnose, which is usually a low-abundance metabolite in plant extracts. (**a**) EI mass spectrum of rhamnose. (**b**,**c**) Peak areas (n = 3), which are indicated for each used concentration of the standard, were integrated from extracted ion chromatograms reconstructed for characteristic values of *m*/*z* = 277 and t_R_ = 23.60–23.80 min for external calibration and t_R_ = 23.50–23.60 min for standard addition calibration. In the external calibration table, the orange and red frames mark rhamnose concentrations, which correspond to the LOD and LOQ, for which S/N is ≥3 and ≥10, respectively. (**d**) External calibration curves built using coordinates of peak areas and standard concentrations and their log-transformed values. (**e**) Standard addition calibration curve. EIC(or XIC)s—extracted ion chromatograms.

Quantification of the analytes in samples is based on the registration of quantitative chromatographic information such as peak areas or peak heights. To establish the relationship between the quantitative characteristics and analyte concentration, calibration curves are constructed by analyzing a series of standard solutions with known concentrations (Figure 3). Analysis of dilution series prepared for individual standards is laborious and time-consuming. Therefore, it is possible to prepare a mixture of known compounds, while observing an important condition for the preparation of such mixes—the t_R_s of the standards should not overlap [88,108]. In addition, when calibrating concentrations of the standards, to reduce possible risk of coelution of two or more standards and their co-occurrence in the same TIC peak, it is recommended to use not the area (height) of the TIC peak, but the peak parameters obtained from an extracted ion chromatogram (XIC), i.e., a chromatogram reconstructed for the analyte-specific *m*/*z* and t_R_ values (Figure 3a–c).

Calibration is a fundamental process used to establish a relationship between the instrumental response and an analyte concentration [120]. This relationship enables accurate quantification and identification of the target analyte. Two common calibration approaches are used for absolute quantitative analysis—external standardization and the standard addition method—each with its own advantages and limitations and suitable for different analytical scenarios [120,121].

In the case of external standardization, a series of calibration solutions with known concentrations of reference compounds (standards) corresponding to the target metabolites of interest is prepared, at least in triplicate. These calibration solutions are then analyzed in the same batch as the plant samples to confirm the identification of metabolites by coelution with the corresponding standards and to construct calibration curves establishing a linear relationship between the standard concentrations and quantitative characteristics (area and height) of the corresponding chromatographic peaks. The calibration curve can be plotted using the coordinates of actual or logarithmic transformed values for peak area (height) and standard concentrations (Figure 3d). While ordinary calibration is suitable for measuring metabolites within a narrow concentration range, log-transformed calibration allows for a more uniform coverage of the multi-order range, considering both low- and high-abundance metabolites in biological samples. It should be noted that the points on the calibration curve should be equidistant from each other, and log-transformed calibration allows this to be achieved. Based on the calibration curves, the LDR, limit of detection (LOD) and limit of quantification (LOQ) for each standard should be determined [117]. LDR indicates the maximum concentration range over which a calibration curve remains linear. The curve linearity is usually assessed by the coefficient of determination, R^2^ [122]. LOD is considered the lowest concentration of an analyte from which it is possible to infer its presence in a sample, and LOQ is the lowest concentration of an analyte from which it is possible to determine it in a sample [123,124]. Since mass spectrometers have a logarithmic response allowing detection at several orders of magnitude of the metabolite concentrations, a calibration curve plotted with log-transformed coordinates in which LDR typically spans multi-orders of magnitude of the metabolite concentrations is often preferred. Therefore, log-transformed calibration helps to align the results with the linear detection range, where the mass spectrometer operates most sensitively and accurately. Thus, log-transformed calibration covers a wide concentration range and improves the accuracy and reliability of metabolite content measurements.

For external standardization, standards are selected that represent the target metabolites of interest and maintain their structural stability at high temperatures under GC-MS conditions. This approach allows determination of the content of target metabolites in samples, and, therefore, comparison of the metabolite levels in samples collected from different experimental conditions, which provides valuable information about the metabolic responses of the object under study [5,99,107].

However, external standardization may not fully account for matrix effects present in the complex plant samples and associated instrument variations, particularly for minor metabolites, leading to potential inaccuracies [97]. To address this issue, the standard addition method is employed [2,102]. In this method, serial dilutions of known quantities of a standard are prepared similarly to the external calibration, but the solvent is an extract of the plant sample (matrix) (Figure 3c). Adding a standard of known concentration to the sample matrix allows a sample-specific calibration curve to be constructed, taking into account the influence of the matrix and associated instrumental variations [97,102]. The LDR obtained from the calibration curves is used to determine the concentrations of the targets in the plant samples (Figure 3e) [89].

Relative quantification is based on a comparison of peak quantitative characteristics (areas and heights) obtained from samples subjected to experimental conditions versus those of control samples. This type of quantification is usually used for those compounds for which pure standards do not exist or are difficult to obtain, as well as for unknown compounds. The relative quantification aims are to distinguish different groups of samples by monitoring changes in their metabolite levels and to identify groups of marker metabolites which are potentially related to the factor impact causing the group differentiation.

## 5. Data Interpretation

Due to the impressive development of MS-based analytical platforms, the analysis of biological samples today generates massive amounts of primary raw data information [125,126]. As each GC-MS run might contain a few hundred features, manual processing of such datasets is not only irrational, it is impossible. Therefore, adequate analysis of the acquired datasets requires the development of software tools for proper data mining and interpretation. These tools need to consider different hardware platforms potentially applicable in metabolomics. Currently, there is a broad selection of commercially and free available programs, which give access to data interpretation in a batch mode with programming and automatization of nearly all steps of the data mining (pre-processing), processing and post-processing (statistical interpretation) (Table 2) [127,128,129].

**Figure 4:**
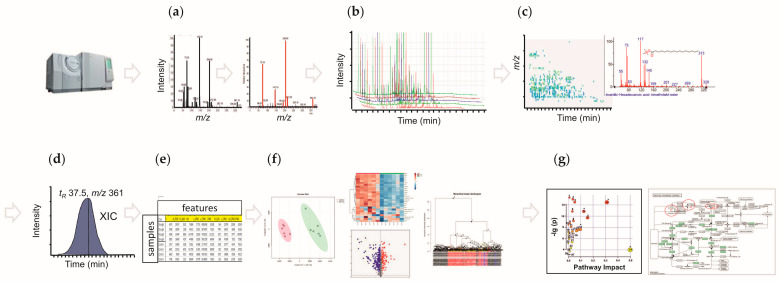
GC-MS data mining and processing workflow. Data mining: (**a**) deconvolution of mass spectra; (**b**) chromatogram alignment by analyte retention times (t_R_); (**c**) peak picking and analyte identification, RI—retention index; (**d**) peak area integration (XIC—extracted ion chromatogram reconstructed for characteristic *m*/*z* values and t_R_s of individual metabolites). Data processing: (**e**) building a matrix of integrated analyte peak areas. Data post-processing: (**f**) statistical analysis; (**g**) elucidation of metabolic pathways.

Data mining in GC-MS-based metabolomics includes the following steps: baseline correction, mass spectrum deconvolution of analytes, peak recognition, peak peaking and chromatogram alignment [130]. This workflow is typically followed by a more or less standardized data processing procedure which is finalized with structural annotation of the aligned features (this is often referred to as identification, although the correctness of this term in this case is questionable [57]) and their further quantification via integration of peak area. Basic steps of data mining, processing and post-processing are shown in Figure 4.

Alignment refers to the procedure of pre-processing and handling datasets comprising both small and large numbers of sample runs. Chromatographic shifts (i.e., variations in t_R_ across the experiment), caused by a change in gas carrier velocity [131], can lead to misalignment of peaks and hinder accurate comparison of all chromatographic peaks, often referred to as features. After completing the alignment, verification is required to confirm that corresponding peaks are aligned in a consistent manner [57,127]. The correctness of the alignment procedure is critically important for identification and quantification of metabolites across the samples and for adequate and reliable statistical analysis. Furthermore, baseline correction is performed to remove baseline drift or fluctuations that may be present in the chromatographic data due to column stationary-phase bleed, background ionization, and low-frequency variations in the detector and/or instrument-controlled parameters (e.g., temperature or gas flow rate) [130]. Baseline correction procedures are specifically designed to correct low-frequency noise and offsets without affecting higher-frequency variations, aiming to enhance the visibility of metabolites and improve the accuracy of peak integration and quantification [132].

There are different methods available to reduce high-frequency noise variations and enhance the quality of chromatograms by improving the S/N ratio. One commonly used technique is smoothing, which involves fitting a low-order polynomial to each data point and its neighbors using the Savitzky–Golay method [133]. Another approach is wavelet smoothing, which transforms the chromatogram into the frequency domain, removes high-frequency noise, and then converts it back to the time domain [134]. These noise reduction techniques play a crucial role in addressing analytical challenges and enabling the discovery of meaningful chemical differences between samples. However, it is important to select the appropriate parameters to avoid distorting the genuine chemical signals and introducing artifacts during the noise removal process [130].

Another critical aspect of the pre-processing pipeline is data cleaning. This assumes the disclosure, identification (if possible), and removal of artifacts, as well as instrumental noise, that may be present in the raw data. Artifacts can arise from various sources, such as sample preparation, instrument performance, or experimental conditions [57]. By carefully checking the data and applying appropriate filtering techniques, these unwanted features (ghost peaks) can be eliminated, thereby improving the accuracy and reliability of the subsequent analysis.

To date, the above-described pipeline is implemented in practice as a part of multiple software solutions. Thus, at least some steps of this workflow or the whole pre-processing pipeline can be accomplished within the XCMS platform [135,136], along with free-access tools like AMDIS (for qualitative analysis) [137], OpenChrom [138], MS-DIAL [139] (for qualitative and quantitative analysis), and others, including commercially available software (Table 2). However, the results need to be manually reviewed, and the instrument-related settings need to be adjusted if necessary. Ideally the processing method needs to be validated with the set of known standards and/or QCs, as described above.

After all data-mining steps have been completed, data processing is performed, starting with structural annotation (identification) of metabolites. It should be noted that the primary benefit of GC-EI-MS is the fact that electron ionization (EI) is a hard ionization technique [23]. Due to this, the fragmentation behavior of individual analytes (both in terms of *m*/*z* patterns and relative intensities of individual signals) is highly reproducible and depends only on electron energy, not on the mass analyzer type or instrument vendor [23,29]. Based on the ionization/fragmentation efficiency distribution of natural products, an electron energy of 70 eV was selected as the standard condition for metabolomics experiments [140]. This setting yields highly informative EI mass spectra, which provide rich structural information about the analyte. This information can be used for structural annotation and, in the best-case scenario, for unambiguous identification of the metabolite. In GC-EI-MS-based metabolomics, this task can be performed by two principal strategies. First, the mass spectra, acquired for the metabolite MeOX-TMS derivatives in the experimental samples, can be compared with those of the corresponding authentic standards (ideally in coelution experiments) to confirm the structural identity of detected analytes [141]. Alternatively, the structures behind the detected features can be annotated by a spectral similarity search, which typically relies on one or several databases—spectral libraries [141,142].

Despite its great impact on the structural annotation of metabolites, mass spectrometry data alone are often insufficient for the identification (i.e., exact and complete structure assignment) of detected compounds, particularly when just minor structural differences between isomers with identical mass spectra need to be distinguished. In this context, a proper annotation of epimeric monosaccharides (e.g., glucose, galactose and mannose, which are typically simultaneously present in plant extracts) is a challenging task, which, obviously, cannot be solved only by interpretation of the mass spectra. Therefore, annotation of such sugars (or other compound classes featuring pronounced isomerism) also relies on other criteria, specifically metabolite-specific t_R_s and Kovats or Fiehn RIs, which describe the retention of substances relative to the series of n-alkanes or FAMEs, respectively [143].

Despite t_R_ providing important information for compound identification, it cannot be considered to be a reproducible parameter as it is strongly amenable to batch effects. Indeed, any instrument- or/and time-specific alteration in various GC-MS conditions (capillary column parameters, column ageing, temperature gradient, etc.) results in changes in the conditions of chromatographic separation and, therefore, in t_R_ shifts. This might directly cause problems with accurate compound identification [144]. To compare the retention behavior of individual compounds observed in GC-MS experiments accomplished in different laboratories under different instrumental conditions or even at different times within the same laboratory, RIs are usually employed.

As mentioned above, t_R_s, RIs and mass spectra together provide important chromatographic and spectral data (GC-MS information) for subsequent analyte annotation. However, to effectively utilize this information, match factors are used to quantify the accuracy of the analyte annotation based on the similarity of the experimental mass spectrum and/or RI to a library reference [141,145,146,147]. Most GC-MS data processing tools (Table 2) have in-built algorithms that determine the match factor, which allows for automatic annotation of analytes based on a comparison with the obtained mass spectra and comparison of RIs with the corresponding available information from in-house or open MS libraries. Among the publicly available MS libraries which contain spectral and RI data for annotation of metabolites, the most widely used are the National Institute of Standards and Technology (NIST; EI mass spectra of ~300,000 compounds, https://webbook.nist.gov/chemistry/ (accessed on 28 November 2025)), the Human Metabolome Data Base [148] (HMDB; ~200,000 entries with EI mass spectra of both lipid- and water-soluble compounds, https://hmdb.ca/ (accessed on 15 January 2022)), MassBank [149] (>90,000 unique EI mass spectra, https://massbank.eu (accessed on 25 November 2025)), and the Golm Metabolome Database [150] (GMD; EI mass spectra of >3000 reference substances and plant metabolites, http://gmd.mpimp-golm.mpg.de/ (accessed on 5 January 2025)); other databases are available as well (Table 2).

**Figure 5:**
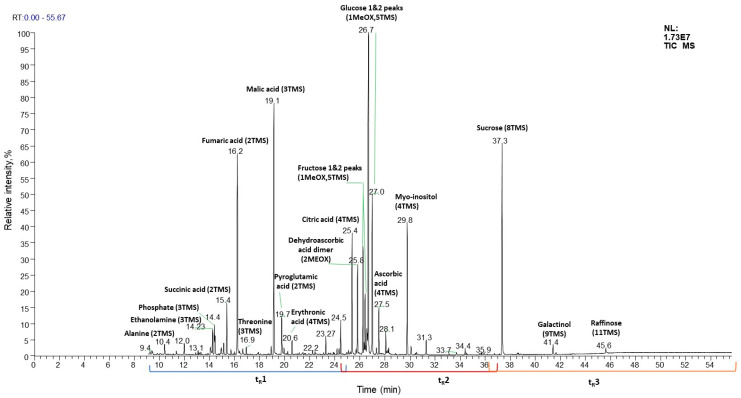
A typical total ion current (TIC) chromatogram obtained from GC-EI-Q-MS analysis of 7-week-old *Arabidopsis thaliana* plants, with marked retention time windows (t_R_ = 1–3) indicating the position of some chemical classes of primary metabolites in the chromatogram. t_R_1—C3–5 organic acids, amino acids (Gly, Ala, Val, Ser, Thr, Pro, Asp, Glu, Asn, Gln, Met, Phe), C3–5 carbohydrates and their derivatives; t_R_2—C6–7-monosaccharides and their derivatives, amino acids (Lys, Tyr, Trp), C16–18 fatty acids; t_R_3—di- and oligosaccharides and their derivatives, sterols, lysolipids.

Despite the advantage of automatic annotation in rapid analyte identification, the results obtained can often be erroneous because the correctness of automatic annotation is highly dependent on the quality of the analyte mass spectrum. Therefore, to improve the reliability of automatic annotation, the analyte spectrum must first be deconvolved to remove noise signals. Then a presumptive annotation should be carefully checked based on the GC-MS information of coeluting reference compounds to establish reliable identification of the analyte. If the GC-MS information of an analyte does not match confidently any coeluted or library reference, the analyte structural annotation (i.e., analyte identification) cannot be established. In this case, it remains possible to perform an annotation to a specific chemical class. The extensive use of trimethylsilylation and EI at 70 eV, which provides the high reproducibility of GC-MS analyses, allowed us to relate the t_R_ of the analyte to its approximate molecular weight and to roughly estimate the possible chemical nature of the analyte depending on the retention time region (t_R_ window) in which the given primary metabolite is located in the chromatogram (Figure 5). To more accurately assign an analyte to a specific chemical group, mass spectrometric information is needed.

TMS derivatives are formed by compounds possessing various functional groups such as alcohols, carboxylic acids, carbonyls (enol-TMS ethers), phosphates, amines, thiols, etc. The elucidation of fragmentation patterns and the mechanisms of formation of the compound groups allows us to specify characteristic (diagnostic) fragments which are present in mass spectra for each of the groups as specific or a few relatively intense signals. For example, the diagnostic fragments for 1TMS and 2TMS amine derivatives are specific signals at *m*/*z* values of 102 and 174 respectively; for TMS ethers of fatty acids, the *m*/*z* values are 132, 117, 145 and 129; for MeOX-TMS C6-aldosaccharides, the *m*/*z* values are 319, 217 and 205; for TMS glycerol-phosphates, the *m*/*z* values are 299, 315 and 445; while for TMS sugar-phosphates, the *m*/*z* values are 299, 315 and 387. The detailed mechanisms of formation of these and other diagnostic fragments characterized by specific *m*/*z* values were reviewed by Harvey and Vouros [151]. The information on diagnostic signals at specific *m*/*z* values together with knowledge of chemical group-specific t_R_ windows allows the analyte to be annotated according to the chemical class. However, if detected analytes cannot be annotated by reference spectra to an individual compound or even to a chemical class, they need to be regarded as unknowns. In this case, their annotation relies on the unique t_R_s, RIs, and EI mass spectra. The patterns of the most characteristic and intense MS signals (characteristic *m*/*z* values of the diagnostic ions) ideally complement RI data for primary annotation of unknowns [29,57,140].

A typical GC-MS run delivers hundreds of annotated features, each of which corresponds to a chromatographic peak [2,21]. Therefore, several features can represent one metabolite (for example, different MeOX-TMS derivatives of the same metabolite) [141]. The intensities of an analyte in different samples can be compared on a relative basis assuming the linear dependence between peak area (or its height) and compound concentration. Therefore, due to the high probability of individual coelutions, TICs are not recommended for peak integration. In contrast, XICs can be reconstructed for characteristic *m*/*z* values (nominal or exact—depending on the instrumental platform used), and the signals of individual metabolites can be integrated at characteristic t_R_ values [29]. The integrated peak areas are used in the further steps of the data processing workflow, which are statistical analysis and biological interpretation of the resultant statistically confirmed data [57,140,152].

When working with data obtained by either targeted or untargeted strategies, the processing step yields a so-called feature quantification matrix (FQM) or biomatrix, which contains the peak areas (heights) obtained from GC-MS data for all features in all experimental samples [57,140,153]. Values for these quantitative characteristics (i.e., peak areas or heights) may be missed because certain compounds may not be detected in some samples. Those missing values may affect further statistical analysis and interpretation of the results. Depending on the reasons for missing values (biological or technical, random or not), various imputation algorithms are applied, such as replacement by mean or median, k-nearest neighbors (kNN), probabilistic PCA (PPCA), Bayesian PCA (BPCA), singular value decomposition (SVD), random forest (RF) and quantile regression imputation of left-censored data (QRILC). All these methods have their advantages and limitations, and the reader is referred to recent reviews on this topic [154,155]. Importantly, before an imputation algorithm is applied, a biomatrix has to be filtered based on the percentage of missing values. This should be accomplished in accordance with the so-called “80% rule”, which sets a threshold on the percentage of missing values for a specific metabolite, such as 20%. If the amount of missing values in the biomatrix exceeds the threshold, then the imputation loses its statistical meaning because it would distort the data matrix and lead to inaccurate results [154]. Therefore, metabolites with a high percentage of missing values have to be excluded from further analysis, or additional experiments have to be conducted to fill in the obtained gaps.

Before the imputation of missing values, the biomatrix is cleaned of intensities of ghost peaks detected in blank samples and normalization is performed. Various normalization strategies are employed in plant metabolomics, such as fresh weight (FW) or dry weight (DW) normalization, which adjusts metabolite levels based on the sample’s fresh or dry weight, helping to account for variations in the doses of samples. Total sum normalization (TSN) and median normalization (MN) are used to adjust the overall intensity of samples, ensuring that the total signal or median of the dataset is consistent across samples [156,157]. Additionally, probabilistic quotient normalization (PQN) accounts for systematic biases by normalizing metabolite levels using ratios between metabolites [158]. After imputation of missing values, data scaling is required to adjust each variable by a factor computed based on the dispersion of the variables, which helps to improve the performance of further statistical analysis [154,158].

At the next post-processing step, univariate and multivariate statistical analysis, along with mathematical modeling, is applied to the multivariate data generated by metabolomics experiments to derive meaningful information and test for significant differences in individual metabolites between different groups [159,160,161]. Several univariate analysis methods are available for metabolomic data analysis. For example, parametric tests like ANOVA, Student’s *t*-tests, and z-tests are commonly applied when comparing differences between two or more groups, provided that assumptions of normality are met [159]. The normality of data can be verified using tests such as the Kolmogorov–Smirnov normality test or Bartlett’s homogeneity of variances test [161]. When normal distribution assumptions are not met, non-parametric methods like the Mann–Whitney *U* test or Kruskal–Wallis one-way analysis of variance can be employed [160]. Moreover, analysis of metabolomic data is considered to apply additional multiple testing. Since many metabolomic properties are examined at once in most investigations, there is a high likelihood of discovering statistically significant results by accident. In order to address this problem, a variety of correction techniques are available, each of which maintains a balance between avoiding false-positive metabolite correlations and preventing the elimination of real linkages (false negatives) [161,162]. One commonly used multiple-test correction method is based on minimizing the false discovery rate (FDR) [163], aiming to minimize the expected proportion of false positives among the total number of positive results, and it has been extensively reviewed by Broadhurst et al. and others [159,161,162].

Multivariate methods consider all features simultaneously, enabling the identification of relationship patterns between them, and can be divided into two groups: supervised and unsupervised methods [159]. Unsupervised methods focus on summarizing complex metabolomic data and identifying data patterns correlated with experimental or biological variables [161]. PCA is the most commonly used unsupervised method in metabolomics. It transforms metabolic features into linearly uncorrelated variables known as principal components, capturing most of the variability in the dataset. Other unsupervised methods like Hierarchical Clustering Analysis (HCA) and Self-Organizing Maps (SOMs) are used to visualize metabolic phenotypes and feature patterns. Supervised methods are usually used when the difference between groups is proved by unsupervised methods. They reduce the impact of additional potential sources of variability when identifying patterns of metabolites associated with the studied phenotypic manifestations because, unlike the unsupervised approach, they use information about the structure of the experiment (i.e., about biological samples belonging to certain variants). Partial least squares (PLS) is a widely used supervised method that can be employed for regression analysis or binary classification (PLS-DA) [164]. Unlike PCA, PLS components focus on the covariance between the variable of interest and the metabolomic data, making it useful for building classifiers based on metabolomic features. Orthogonal PLS (O-PLS) was developed to address the problem of unrelated metabolic features influencing results [164].

The analysis of metabolite networks is the last step in GC-MS-based plant metabolomics studies, providing valuable insights into the dynamics and functional significance of the metabolome within the biological system under investigation [88,128,165]. This step involves the exploration and interpretation of interconnected relationships between metabolites using various computational and statistical approaches based on the information of plant networks from the databases. Techniques like correlation networks and metabolic pathway analysis are commonly employed at this step, and advanced methods like graph theory allow us to quantify the importance and connectivity of individual metabolites [166,167]. To assist with the data analysis process, we have access to online platforms such as MetaboAnalyst [168] and Visual Analysis and Exploration of Networks containing Experimental Data (VANTED) [169], which offer statistical and pathway analysis tools specifically designed for metabolomic data. These platforms enable normalization, clustering, and modeling using various algorithms. Additionally, there are other resources available for metabolomics data analysis, as indicated in Table 2. The application of machine learning has become increasingly relevant in interpreting metabolomics data, contributing to unraveling complex relationships, and it provides opportunities for targeted metabolic engineering, biomarker discovery, and crop improvement [170,171].

**Table 2:** A few databases and tools for GC-MS data analysis in metabolomics studies.

Name of the Tool/Database	Brief Description	Website URL (Accessed on 1 September 2025)	Ref.
ADAP (Automatic Data Analysis Pipeline)	A tool that provides a range of advanced algorithms and statistical methods for data analysis.	http://www.du-lab.org/software.html	[172]
AMDIS (Automated Mass Spectral Deconvolution and Identification System)	A tool used for spectral deconvolution and metabolite identification from GC-MS data.	https://chemdata.nist.gov/dokuwiki/doku.php?id=chemdata:amdis	[173]
MeltDB 2.0	A comprehensive online platform for metabolomics data management and analysis. It offers a range of tools to facilitate the storage, processing, and interpretation of metabolomics data.	https://meltdb.cebitec.uni-bielefeld.de/cgi-bin/login.cgi	[174]
MetaboAnalyst	A comprehensive web-based tool for metabolomic data analysis and interpretation, offering various modules for statistical analysis, data preprocessing, pathway analysis, and visualization.	http://www.metaboanalyst.ca/	[168]
MetaboLights	A web-based repository and analysis platform for metabolomics data which serves as a comprehensive resource for researchers to store, share, and analyze metabolomics datasets.	https://www.ebi.ac.uk/metabolights/	[175]
MetaboliteDetector	The software offers a comprehensive and automated data analysis pipeline, starting from raw GC-MS data and culminating in principal component analysis.	https://md.tu-bs.de/	[176]
MetAlign	A tool offering a range of features and algorithms to facilitate accurate peak detection, alignment, and normalization of metabolite signals across multiple samples.	https://zenodo.org/record/7273832	[177]
metaX	A comprehensive tool for processing and post-processing mass spectromrtry data.	https://metax.genomics.cn/	[178]
MS-DIAL	A comprehensive software tool developed for the analysis of mass spectrometry data, particularly in metabolomics studies.	http://prime.psc.riken.jp/compms/msdial/main.html	[139]
MET-IDEA (Metabolomic Identification by Database Enrichment Analysis)	A bioinformatics tool designed for the identification and annotation of metabolites in GC-MS-based metabolomics studies.	http://www.msea.ca	[179]
Mzmine	A software tool for processing, visualization, and analysis of MS-based metabolomics data.	https://mzmine.github.io/	[167]
OpenChrom	An open-source software platform designed for GC-MS data analysis, providing a range of powerful features for processing and visualizing chromatographic data.	https://www.openchrom.net/	[138]
SIMAT	A comrehensive tool for analysis of GC-MS data acquired in SIM mode.	https://omics.georgetown.edu/tools#h.72n4e5rnc2eg	[180]
PyMassSpec	A Python (PyMass 2.5.0) library designed for mass spectrometry data analysis. It provides a comprehensive set of tools and functions for processing, analyzing, and visualizing mass spectrometry data.	https://pymassspec.readthedocs.io/en/master/	[181]
TagFinder	A specifically designed tool for the identification and quantification of volatile and semivolatile compounds in complex biological samples.	https://www.mpimp-golm.mpg.de/19405/Corrector_package_V1_91.zip	[182]
XCMS	A software package for processing and visualizing MS-based metabolomic data. It offers various algorithms and methods for peak picking, retention time alignment, background subtraction, and normalization.	https://xcmsonline.scripps.edu/	[135]
Commercially available tools			
AnalyzerPro	A comprehensive software tool designed for the analysis of GC- and LC-MS data in metabolomics research, offering a range of advanced features to process, visualize, and interpret metabolomic data.	https://spectralworks.com/software/analyzerpro/	
ChromaTOF	Software specifically designed to process and analyze raw GC-MS data, providing a range of features for data processing, peak detection, compound identification, and data visualization.	https://www.leco.com/product/chromatof-software	
QuanLynx	The software provides advanced data processing capabilities, including peak picking, alignment, quantification, and statistical analysis.	https://www.waters.com/waters/library.htm?locale=en_US&lid=1545661	
Progenesis QI	Software for peak detection, alignment, and normalization used to accurately quantify metabolites in complex samples that provides a range of statistical tools for differential analysis, multivariate analysis, and pathway analysis.	https://www.waters.com/waters/en_US/Progenesis-QI-Software/nav.htm?cid=134790655	
Xcalibur	The software provides a comprehensive set of tools for data acquisition, instrument control, data processing, and analysis.	https://www.thermofisher.com/order/catalog/product/OPTON-30965	
MassLynx	Offers a user-friendly interface with a range of features for data acquisition, instrument control, data processing, and analysis.	https://www.waters.com/waters/en_US/MassLynx-MS-Software/nav.htm?locale=en_US&cid=513662	
Databases and pathway-related tools			
BioCyc	A collection of curated databases that provides comprehensive information on the genomes and metabolic pathways of various organisms.	http://biocyc.org/	[183]
HMDB (Human Metabolome Database)	A comprehensive metabolomics and biochemistry database, enabling searches for metabolites, pathways, chemical structures and biological functions.	https://hmdb.ca/	[148]
KEGG (Kyoto Encyclopedia of Genes and Genomes)	A bioinformatics resource that integrates genomic, chemical, and systemic functional information. It provides a comprehensive collection of biological pathways, genomic information, and functional annotations for various organisms.	http://www.genome.jp/kegg/	[184]
MapMan	A tool designed for the visualization and analysis of omics data, with a particular focus on plant systems, providing a comprehensive mapping and annotation platform that allows interpretation and exploration of high-throughput data in the context of metabolic pathways and cellular processes.	http://mapman.gabipd.org/	[185]
MetaCrop	A comprehensive database focusing on the metabolism and pathways of crop plants.	http://metacrop.ipk-gatersleben.de	[186]
MetaMapp	A comprehensive bioinformatics tool designed for the integrated analysis of metabolomic and transcriptomic data.	http://metamapp.fiehnlab.ucdavis.edu/	[187]
MetNetDB	A comprehensive metabolomics database and analysis platform that integrates metabolite data with biochemical pathways and regulatory networks.	https://metnetweb.gdcb.iastate.edu/	[188]
MetiTree	Prototype repository of mass spectra of small chemical compounds for life sciences (<2000 Da).	http://www.metitree.nl/	[189]
MetScape	A bioinformatics tool designed for the visualization and interpretation of metabolomics and transcriptomics data in the context of metabolic pathways.	http://metscape.ncibi.org/	[190]
Pathvisio	An open-source pathway visualization tool for the analysis of omics data integration.	http://www.pathvisio.org/	[191]
PathWhiz	Web-based tool for visualizing and exploring biological pathways.	https://smpdb.ca/pathwhiz	[192]
PMN (Plant Metabolic Network)	Comprehensive resource for plant metabolomics research, including a curated collection of plant metabolic pathways and functional annotations.	http://www.plantcyc.org/databases	[193]
TAIR (The Arabidopsis Information Resource)	A comprehensive online database dedicated to the model plant species *Arabidopsis thaliana*.	https://www.arabidopsis.org/index.jsp	[194]
GMD (The Golm Metabolome Database)	A comprehensive resource that provides a repository of metabolite spectral profiles of a wide range of metabolite classes from various organisms.	http://gmd.mpimp-golm.mpg.de/Default.aspx	[150]
VANTED (Visual Analysis and Exploration of Networks containing Experimental Data)	A comprehensive software tool for the statistical analysis, visualization and analysis of biological networks, including metabolic pathways.	https://immersive-analytics.infotech.monash.edu/vanted/	[195]
RIKEN Plant Metabolome MetaDatabase (RIKEN PMM)	A comprehensive online resource focusing on plant metabolomics data, provides a curated collection of metabolite information, chemical structures, mass spectra, experimental data, metabolite profiles, metabolic pathways, and statistical analysis.	http://metabobank.riken.jp/pmm/db/plantMetabolomics	[196]

## 6. Concluding Remarks

Over two decades after the pioneering studies of Roessner and Fiehn [1,2], which bridged the gap between plant phenotypes and genotypes, GC-MS in profiling plant compounds has become the method of choice for investigating how plants respond to environmental factors and biotic interactions. Its exceptional separation capabilities, compatibility with complex plant metabolomes, and robustness due to stable derivatives have solidified its role in plant metabolomics. Furthermore, the electron ionization spectra of derivatives contribute to the success of this approach by providing confident identification and sensitive quantification of metabolites. Additionally, the emergence of more accurate analytical platforms, coupled with the power of bioinformatics tools and machine learning, has reduced the time required for data analysis and interpretation, deepening our insights into metabolic networks. The lack of a universal approach to the study of primary metabolites (due to their great diversity) opens up opportunities for experiments at the stages of extraction and separation of molecules. However, quality assessments must be considered at the experimental design stage, notably for the validation of the matrix effect on yields in such groups of compounds as saccharides, sugar phosphates, and organic and amino acids, and the impact of derivatization on artifact occurrence in the analysis of primary plant metabolites. As we move forward, continuous advancements in sample preparation, analytical techniques, and data processing will further enhance the potential of GC-MS in deciphering the intricate metabolic networks of plants. This methodology will undoubtedly continue to be an invaluable tool for enriching our comprehension of plant metabolism and the specific roles of metabolites in various physiological processes.

## Figures and Tables

**Table 1:** Types of mass analyzers applied in GC-MS-based plant metabolomics.

Analytical Platform ^a^	Basic Principle of Mass Analysis	Mass Accuracy (a.m.u.) ^b^	Main Field of Application	Field of Potential Application
Quadrupole	Selective mass filtering based on applied RF and DC voltages	0.1–0.01	Analysis of specific compounds or classes of metabolites	Quantification and confirmation of known metabolites
Quadrupole Ion Trap (QIT)	Ion trapping and mass analysis based on the stability of ions in a quadrupole field	0.1–0.01	Structural elucidation and fragmentation analysis	Screening for unknown metabolites, natural product discovery
Linear Ion Trap (LIT)	Ion trapping and mass analysis based on the stability of ions in a linear RF field	0.1–0.01	MS^n^ experiments, enhanced structural characterization	Identification of isomeric compounds
Quadrupole–Linear Ion Trap (QLIT)	Combination of quadrupole and linear ion trap analyzers for improved selectivity and sensitivity	0.1–0.01	Analysis with enhanced sensitivity and dynamic range	Metabolite pathway analysis, drug metabolite profiling
Time-of-Flight (TOF)	Measurement of ion flight times to determine their *m*/*z*	0.01–0.001	Untargeted metabolomics, comprehensive profiling	Screening for unknown metabolites, biomarker discovery
Quadrupole–Time-of-Flight (Q-TOF)	Combination of quadrupole and TOF analyzers for precursor ion selection and accurate mass measurement	0.01–0.001	Metabolite identification and characterization	Comparative metabolomics, pathway analysis
Magnetic Sector	Deflection of ions based on their *m*/*z* in a magnetic field	0.001–0.0001	Accurate quantification and structural elucidation of metabolites	Isomer differentiation, metabolite profiling
Triple Quadrupole (QqQ)	Selective mass filtering and fragmentation in multiple stages	0.001–0.0001	Quantitative analysis with high sensitivity and selectivity	Metabolite quantification, trace-level analysis
Orbitrap	Detection of ion oscillations in a high-resolution electrostatic field	0.0001–0.00001	High-resolution accurate mass analysis, metabolite profiling	Discovery of unknown metabolites, metabolite annotation
Quadrupole–Orbitrap (Q-Orbitrap)	Combination of quadrupole and Orbitrap analyzers for precursor ion selection and high-resolution accurate mass measurement	0.0001–0.00001	Comprehensive metabolite profiling and identification	Metabolomics, metabolite biomarker discovery

^a^ Platforms are ranged based on their resolution capacity; ^b^ mass accuracy values provided are approximate ranges and may vary depending on the specific instrument setup, calibration, and experimental conditions.

## Data Availability

No new data were created or analyzed in this study.

## Abbreviations

(TMS)_3_Si^•^	Trimethylsilyl radical
BPCA	Bayesian PCA
BSA	*N*,*O*-bis(trimethylsilyl)acetamide
BSTFA	*N*,*O*-Bis(trimethylsilyl)trifluoroacetamide
DLLME	Dispersive liquid–liquid microextraction
DW	Dry weight
EI	Electron ionization
FAMEs	Fatty acid methyl esters
FDR	False discovery rate
FQM	Feature quantification matrix
FTMS	Fourier transform mass spectrometer
FW	Fresh weight
GC×GC	Tandem (two dimensional) gas chromatography
GC-EI-MS	Gas chromatography–electron impact mass spectrometer
GC-EI-Q-MS	Gas chromatography–electron impact–quadrupole mass spectrometer
GC-MS	Gas chromatography–mass spectrometry
HCA	Hierarchical Clustering Analysis
IS	Internal standard
kNN	k-nearest neighbours
LDR	Linear dynamic range
LLE	Liquid–liquid extraction
LLME	Liquid–liquid microextraction
LOD	Limit of detection
LOQ	Limit of quantification
MAE	Microwave-assisted extraction
MBTFA	*N*-methyl-bis(trifluoroacetamide)
MeOX	*O*-methylhydroxylamine hydrochloride
MEPS	Microextraction by packed sorbent
MN	Median normalization
MRM	Multiple reaction monitoring
MS/MS	Tandem mass spectrometry
MS^n^	Multistage mass spectroscopy
MSPD	Matrix solid-phase dispersion
MSPE	Magnetic SPE
MSTFA	*N*-Methyl-*N*-(trimethylsilyl)trifluoroacetamide
MTBSTFA	*N*-tert-butyldimethylsilyl-*N*-methyltrifluoroacetamide
O-PLS	Orthogonal PLS
PCA	Principal component analysis
PLE	Pressurized liquid extraction
PLS	Partial least squares
PLS-DA	Partial least squares discriminant analysis
PPCA	Probabilistic PCA
PQN	Probabilistic quotient normalization
PT-SPE	Pipette-tip SPE
QCs	Quality control samples
QqQ-MS	Triple quadrupole–mass spectrometer
QRILC	Quantile regression imputation of left-censored data
Q-TOF-MS	Quadrupole–time-of-flight mass spectrometer
RF	Random forest
RIs	Retention indices
S/N	Signal-to-noise ratio
SBSE	Stir-bar sorptive extraction
SDME	Single-drop microextraction
SFE	Supercritical fluid extraction
SHWE	Super-heated water extraction
SIM	Selected ion monitoring
SLE	Solid–liquid extraction
SOMs	Self-Organizing Maps
SPE	Solid-phase extraction
SPME	Solid-phase microextraction
SRM	Selected reaction monitoring
SVD	Singular value decomposition
TBDMS	Tert-butyl(dimethyl)silyl
TIC	Total ion chromatogram
TMSA	*N*-trimethylsilylacetamide
TMSI	*N*-trimethylsilylimidazole
TOFMS	Time-of-flight mass spectrometer
TSN	Total sum normalization
UAE	Ultrasound-assisted extraction
XICs	Extracted ion chromatograms
